# Conservative recovery and replacement of a ruptured percutaneous endoscopic gastrostomy tube; a case report

**DOI:** 10.1186/s12876-020-01218-x

**Published:** 2020-03-30

**Authors:** Maan Al Halabi, Wakim Wakim, Hicham Moukaddam, Ahmad Husari

**Affiliations:** 1 Department of Internal Medicine, American University of Beirut, Beirut, Lebanon; 2 Department of Diagnostic Radiology, American University of Beirut, Beirut, Lebanon; 3 Division of Pulmonary and Critical Care Medicine, Department of Internal Medicine, American University of Beirut, Beirut, Lebanon; 4 Division of Pulmonary and Critical Care Medicine, Department of Internal Medicine, American University of Beirut, P.O. Box 11-236, Riad El Solh, Beirut, 1107 2020 Lebanon

**Keywords:** Percutaneous endoscopic gastrostomy, Feeding tube rupture, Peritonitis, Interventional radiology

## Abstract

**Background:**

Percutaneous Endoscopic Gastrostomy (PEG) feeding tubes are frequently placed in patients to provide enteral nutrition. We report a case of a complete rupture of a PEG tube intra-abdominally with associated peritonitis after more than a month of PEG placement and utilization. To our knowledge, this is a very rare case of a complete PEG rupture with the succeeding replacement and recovery of the fractured segments conservatively.

**Case presentation:**

A 69-year-old female with a PEG in position and in use for more than a month started complaining of severe abdominal pain. Digital subtraction angiography (DSA) tubogram revealed rupture and separation of the PEG tube into two fragments.

Interventional radiology (IR) team was successful with their conservative approach. Both fragments were removed conservatively without the need for laparotomy. The distal fragment was utilized to place a guide wire, and a new PEG was placed in position with no intraabdominal leak.

**Conclusion:**

Ruptured PEG tube should be considered in the differential of patients complaining of sudden abdominal pain, especially after chronic PEG utilization. Conservative approach by IR is a viable option in correcting this mishap.

## Background

Percutaneous Endoscopic Gastrostomy (PEG) feeding tube placement is frequently performed to provide continuing nutritional support for a variety of medical conditions, particularly patients with neurologic weakness and difficulty in swallowing. Since its introduction in 1980, PEG placement is becoming “the procedure of choice” as it is a convenient and a non-surgical procedure that is performed under local anesthesia [1, 2]. In general, PEG is considered a relatively safe procedure because it is infrequently associated with minor complications. Very rarely, however, major life-threatening complications like peritonitis do occur [3]. In our patient, we do report a complete intraabdominal rupture of the PEG tube with associated abdominal peritonitis. The ruptured tube was conservatively recovered and replaced by interventional radiology. The patient subsequently recovered and was discharged from the hospital.

## Case presentation

**Fig. 1:**
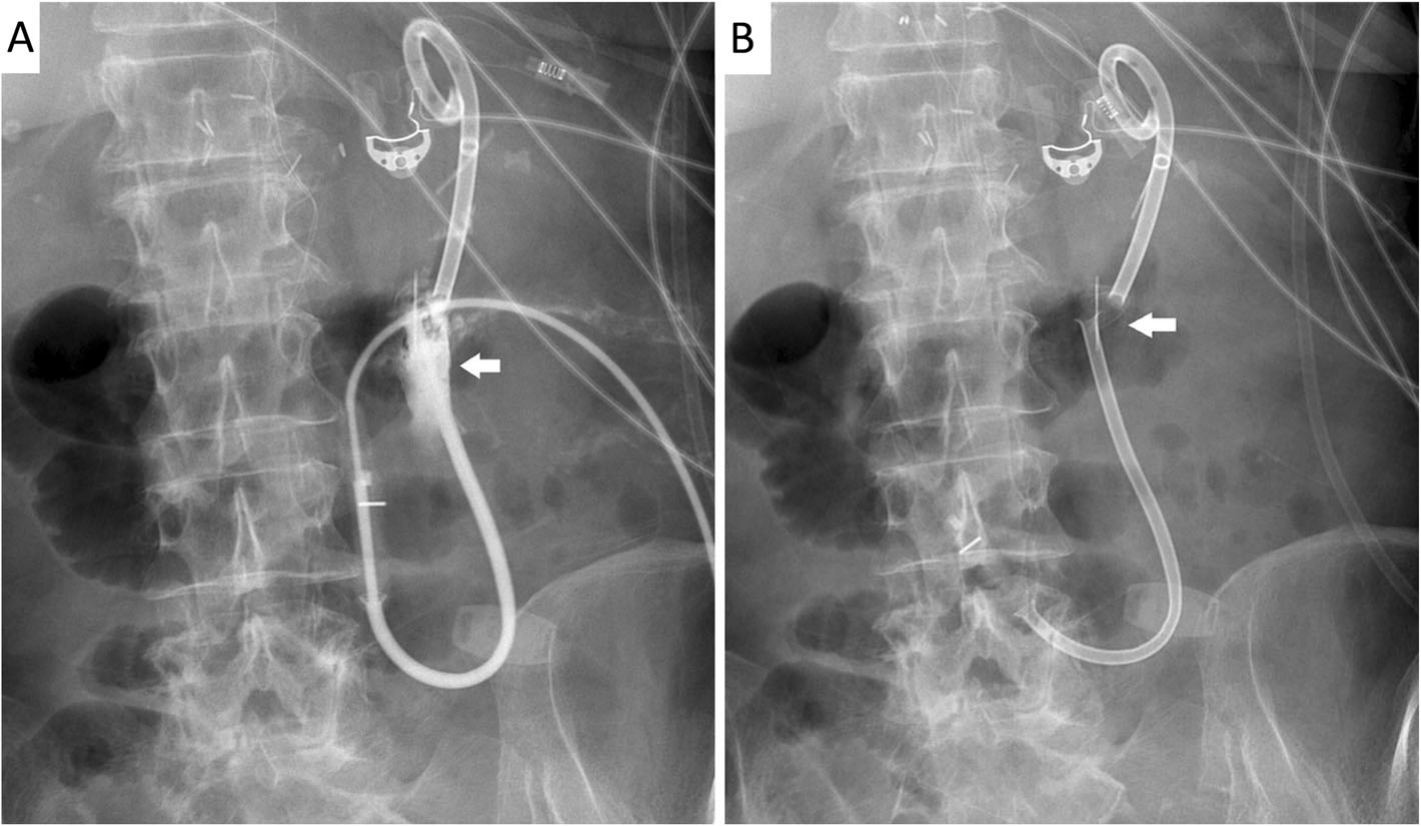
A completely fractured feeding tube is clearly observed (arrow) and water-soluble contrast leak into the peritoneal cavity is noted (**b**)

A 69-year-old female with multiple comorbid medical conditions underwent an emergency repair of an aortic dissection and suffered from prolonged hospital course postoperatively. Because of failure to wean and generalized weakness, tracheostomy and percutaneous endoscopic gastrostomy (PEG) tubes were placed, and both tubes functioned adequately after placement. PEG tube was utilized for feeding and PO meds and continued to function well for more than a month after placement except for the occasional episodes of tube blockage. Tube blockages were resolved by forcefully flushing the tube via a five-cc syringe. On Day 32 post-PEG placement, the patient started complaining of severe abdominal pain. Digital subtraction angiography (DSA) tubogram revealed contrast leak into the peritoneal space and a complete rupture and separation of the PEG tube into a proximal and a distal segment (Fig. 1). CT abdomen demonstrated a significant increase in the density of the ascitic fluid, confirming the clinical diagnosis of peritonitis, and the patient was placed on broad-spectrum antibiotics.

**Fig. 2:**
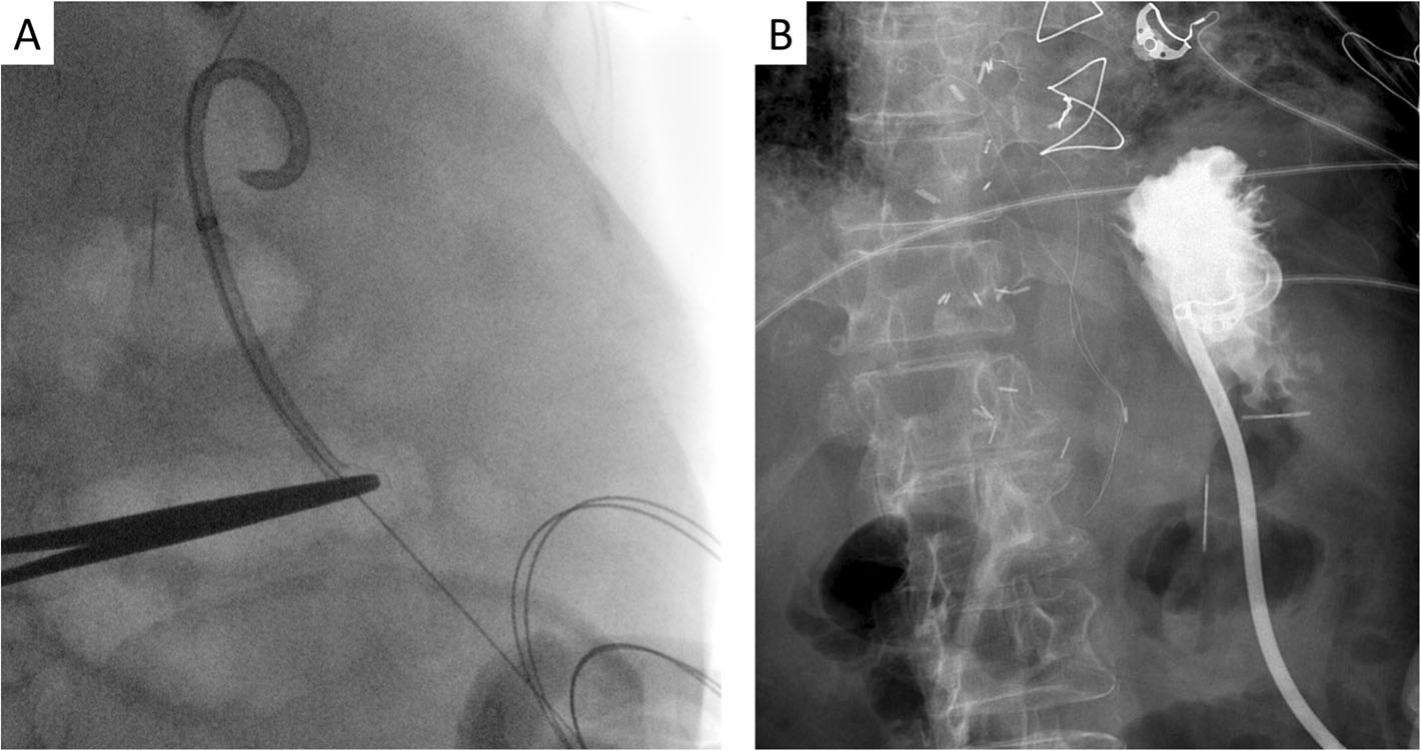
Retrieval of the distal segment of the fractured PEG tube and recanalization using a guide wire. A newly PEG tube is inserted in position, and no contrast leak is detected following the exchange

**Fig. 3:**
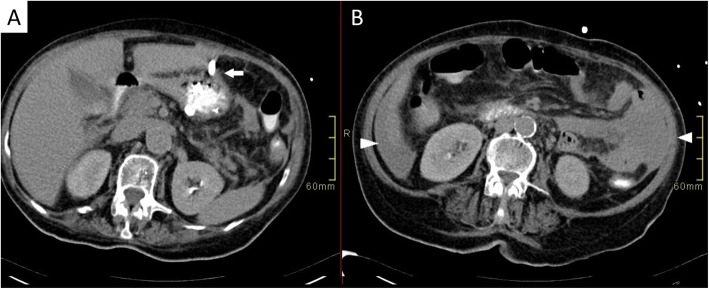
CT scan of the abdomen was performed 24 h after the placement of the new PEG demonstrated no water-soluble contrast leak from the newly placed PEG tube (Fig. 3 **a**: white arrow). Small volume ascites and limited peritonitis is noted secondary to the removed ruptured PEG tube (Fig. 3**b**: arrow tip)

Interventional radiology quickly removed the proximal fragment with no difficulty. Utilizing the locking threads that are part of the PEG apparatus, the distal fragment was conservatively maneuvered and pulled it to the level of the skin of the abdomen (Fig. 2). When the distal fragment became visible at the stoma site, a clamp was placed to secure it in place, a guide wire was then threaded to regain access to the stomach, and a new gastrostomy tube was re-introduced over the guide wire (Fig. 2). Water-soluble contrast was injected, and the location was confirmed with no leaks detected (Fig. 3). The patient subsequently did well, recovered from peritonitis, and transferred to a chronic care medical facility.

## Discussion and conclusions

Percutaneous endoscopic gastrostomy (PEG) is considered a safe modality for providing enteral nutrition to patients who cannot be fed orally and require long-term nutrition. The procedure can be associated with several complications that include site infection and irritation, buried bumper syndrome, stomach ulceration, PEG site leak, and gastric outlet obstruction [3]. PEG tube dislodgement does occur in 2–3% of patients, but it usually occurs within days of placement and can pose a unique clinical challenge [4]. Management usually depends on the duration of dislodgement and the maturity of the PEG tract. Surgical exploration is indicated if signs of sepsis or peritonitis ensue [5, 6].

Chemical peritonitis was the presenting feature in our case that led to the discovery of the fractured PEG tube. It occurred after more than a month of PEG tube placement and utilization. Our patient was managed conservatively without the need for surgical laparotomy. We were lucky to be able to remove the distal segment of the fractured tube by holding to the “locking threads” that are part of the PEG tube. The threads were utilized to fetch the tube to the skin surface, and the fractured tube was also utilized to place a new guide wire and a new PEG tube. Potential causes of a PEG rupture are inappropriate flushing or a defective product. An extensive review of the utilization of the PEG tube in our patient failed to reveal any unusual manipulation like foreign body or hardware insertion. Tube blockages were managed, similar to other patients, by forcefully Flushing the tube utilizing a five-cc syringe. We do not think the pressure generated by a five-cc syringe would lead to tube rupture. The local distributor was informed, and we continue to use the same product with no additional or new observations noted in other patients.

In conclusion and to our knowledge, this is a rare reported case of PEG tube rupture. The case underscores the need to consider fractured PEG tube as part of the differential of patients presenting with peritonitis, especially after chronic PEG placement and utilization. The study stresses the need to test PEG tubes for physical integrity and to create standard recommendations for handling tube blockages. The conservative approach by IR can be successful and should be considered, and the usefulness of the locking threads in the PEG tube apparatus cannot be overemphasized.

Consent section: Written informed consent was obtained from the patient for publication of this case report and any accompanying images.

## Data Availability

Data and Materials will be available from the corresponding author.

## Abbreviations

PEG: Percutaneous Endoscopic Gastrostomy
DSA: Digital subtraction angiography
IR: Interventional radiology
